# Fucci-G1 insertion partially disrupts *Shh* expression

**DOI:** 10.1242/bio.062386

**Published:** 2026-08-04

**Authors:** Yuka Morikawa, Ann Bromley, Jeffrey D. Steimle, James F. Martin

**Affiliations:** ^1^ Cardiomyocyte Renewal Lab, Texas Heart Institute at Baylor College of Medicine, Houston, TX 77030, USA; ^2^ Department of Integrative Physiology, Baylor College of Medicine, Houston, TX 77030, USA; ^3^ Center for Organ Repair and Renewal, Baylor College of Medicine, Houston, TX 77030, USA

**Keywords:** Fucci, *Shh*, Hedgehog signaling, Transgenic mouse model, Genotyping

## Abstract

The Fucci mouse lines are valuable tools for tracing cell cycle stages during development and in postnatal tissues; however, the insertion loci for the transgenes remain unidentified. Here, we report the genomic insertion sites of both the Fucci-G1 and -S/G2/M transgenes, identified through whole-genome sequencing. Interestingly, the Fucci-G1 transgene is located proximal to the *Shh* lung gut enhancer (SLGE) on chromosome 5. Homozygous Fucci-G1 embryos are perinatal lethal but survive until embryonic day (E)18.5, exhibiting craniofacial and foregut-related defects. Expression of *Shh* and canonical targets are downregulated in the pharyngeal arches of E11.5 homozygous Fucci-G1 embryos. Furthermore, *in situ* hybridization analysis showed diminished *Shh* expression in the pharyngeal and foregut endoderm, consistent with disruptions to SLGE activity. Our results indicate that heterozygous Fucci-G1 mice are suitable for cell cycle studies; however, we caution potential genetic interference when investigating SHH-related pathways.

## Teaser

**Summary:** Investigation of Fucci transgene insertion sites uncovered unintended disruption of *Shh* expression and perinatal lethality, providing necessary context for the accurate use and interpretation of this cell-cycle labeling tool.

## INTRODUCTION

The Fucci mouse lines, Fucci-G1 and Fucci-S/G2/M [B6.Cg-Tg(FucciS/G2/M)#504Bsi and B6.Cg-Tg(FucciG1)#596Bsi] (Sakaue-Sawano et al., 2008) have been used for cell cycle staging in developmental, postnatal, and adult tissues (Sakaue-Sawano et al., 2008; Baniol et al., 2021; Hashimoto et al., 2014; Juuri et al., 2012). Although several more Fucci lines with targeted insertions have been established (Zielke and Edgar, 2015), the original transgenic lines are widely used and cited. Despite widespread usage, the insertion loci of neither transgene have been published. It is reported that homozygous Fucci-S/G2/M mice are viable and fertile, and homozygous Fucci-G1 mice are probably embryonic lethal (https://knowledge.brc.riken.jp/resource/animal/card?brc_no=RBRC02707&__lang__=en) with an unknown phenotype. The primary goal of this work is to identify the location of both transgenes to facilitate efficient breeding and genotyping of Fucci mice.

## RESULTS AND DISCUSSION

**Fig. 1.**
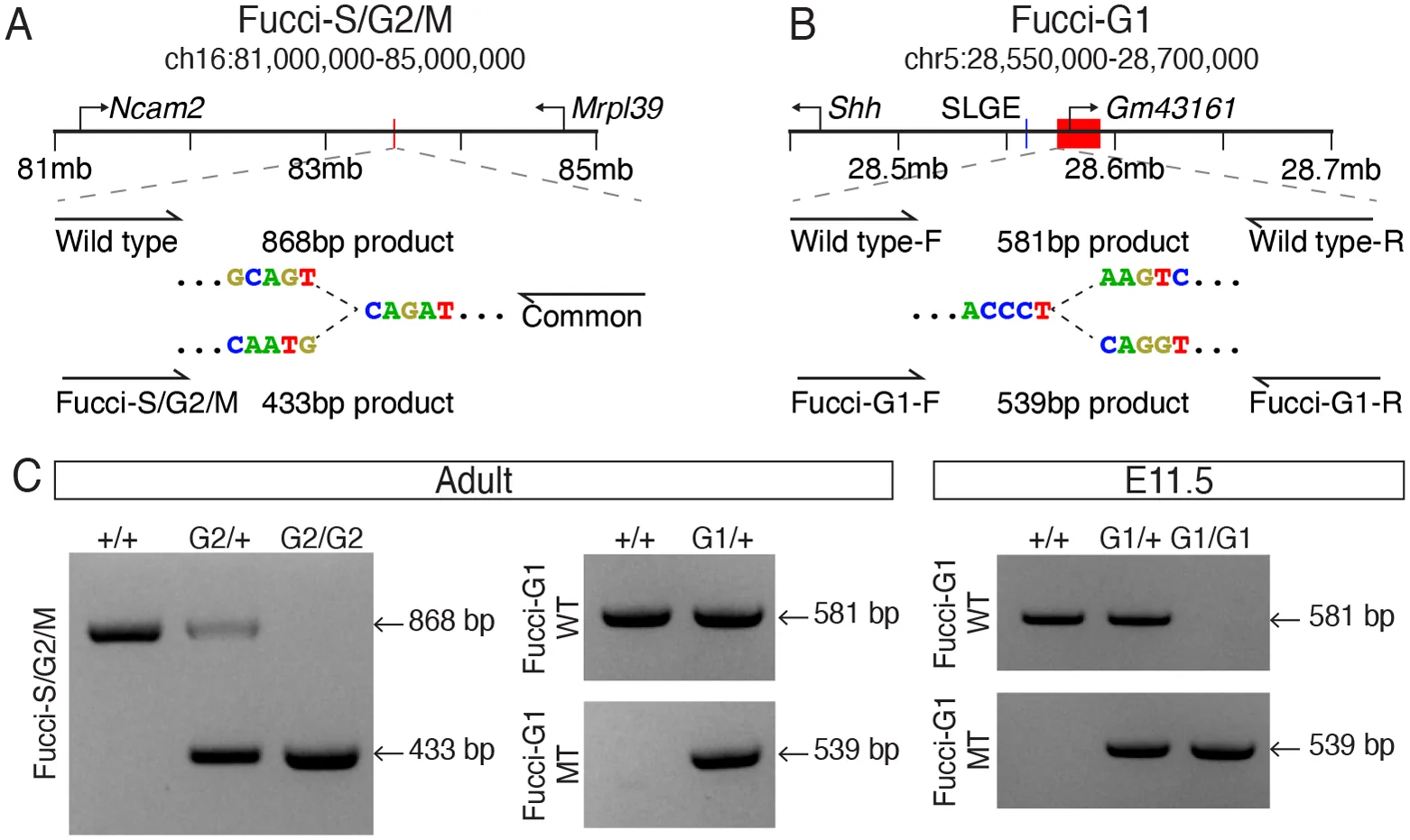
**Identification of Fucci-S/G2/M and Fucci-G1 transgene insertion loci and PCR genotyping with flanking regions.** (A,B) Localization of the Fucci-S/G2/M (A) and Fucci-G1 (B) transgenes. Fucci-S/G2/M inserted on the promoter end at chr16:83,618,748 (mm10), while Fucci-G1 inserted terminal end at chr5:28,573,077 and promoter end at chr5:28,593,624. Predicted PCR product sizes are indicated each primer pair (Materials and Methods). (C) PCR genotyping indicates the positive identification of wild type (+/+), heterozygous Fucci-S/G2/M (G2/+) or heterozygous Fucci-G1 (G1/+), and homozygous Fucci-S/G2/M (G2/G2) or homozygous Fucci-G1 (G1/G1) in adults or E11.5 embryos. WT, wild type.

We conducted whole-genome sequencing on a Fucci-G1 and -S/G2/M double transgenic mouse. We identified one arm of Fucci-S/G2/M inserted into chromosome 16 at 83,618,749 (mm10), a long terminal repeat, approximately 1.2 mb downstream of *Mrpl39* and 1.9 mb downstream of *Ncam2* (Fig. 1A). Fucci-G1 is located on chromosome 5, approximately 100 kb upstream of the *Shh* gene, and its insertion resulted in the deletion of a 20.5 kb region encompassing chr5:28,573,078-28,593,623 (mm10), which includes long intergenic non-coding RNA (lincRNA) *Gm43161* (Fig. 1B).

Based on sequences around the insertion sites, we designed primers to distinguish wild-type, heterozygous, and homozygous mice by PCR genotyping (Fig. 1C, Materials and Methods). As previously reported, we were able to obtain homozygous Fucci-S/G2/M adult mice. However, we could not obtain homozygous Fucci-G1 adults or pups. By genotyping embryos, we were able to obtain homozygous Fucci-G1 embryos at embryonic day (E)11.5 (Fig. 1C). Subsequent analysis shows that embryos survive until E18.5, suggesting that Fucci-G1 homozygotes are perinatal lethal.

**Fig. 2.**
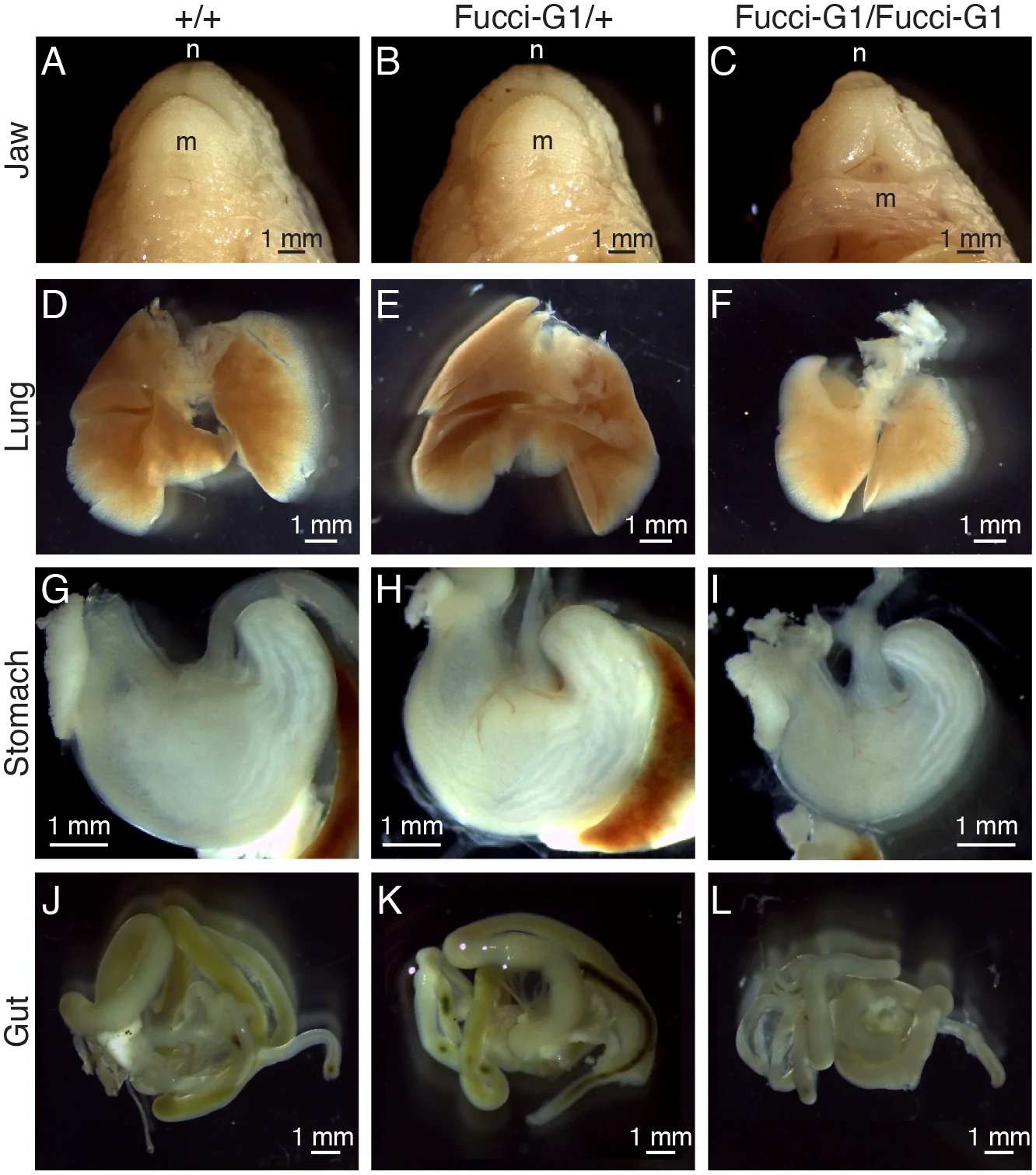
**Fucci-G1 homozygous embryos show hypoplastic organs.** (A-L) Gross morphology of wild type (+/+; A,D,G,J), Fucci-G1 heterozygous (Fucci-G1/+; B,E,H,K), and Fucci-G1 homozygous (Fucci-G1/Fucci-G1) embryos (C,F,I,L) at E18.5. For this experiment, three homozygous, ten heterozygous, and six wild-type embryos were examined. m: mandible, n: nose.

Activation of *Shh* during development is regulated by long-range enhancer elements (Kane et al., 2022). One of the enhancers, *Shh* lung gut enhancer (SLGE) is located 9 kb from the Fucci-G1 insertion site (Anderson et al., 2014; Tsukiji et al., 2014), leading us to hypothesize Fucci-G1 insertion disrupts SHH-dependent development. Fucci-G1 homozygous embryos at E18.5 exhibited hypoplastic jaws, lungs, stomachs, and guts (Fig. 2), all regions of *Shh* expression during development (Litingtung et al., 1998). Several pups were collected immediately following birth at postnatal day (P)0, and persistent phenotypes were observed. Analyses of other organs of Fucci-G1 homozygous late-stage embryos and neonates were comparable to those of wild type at a gross level.

**Fig. 3.**
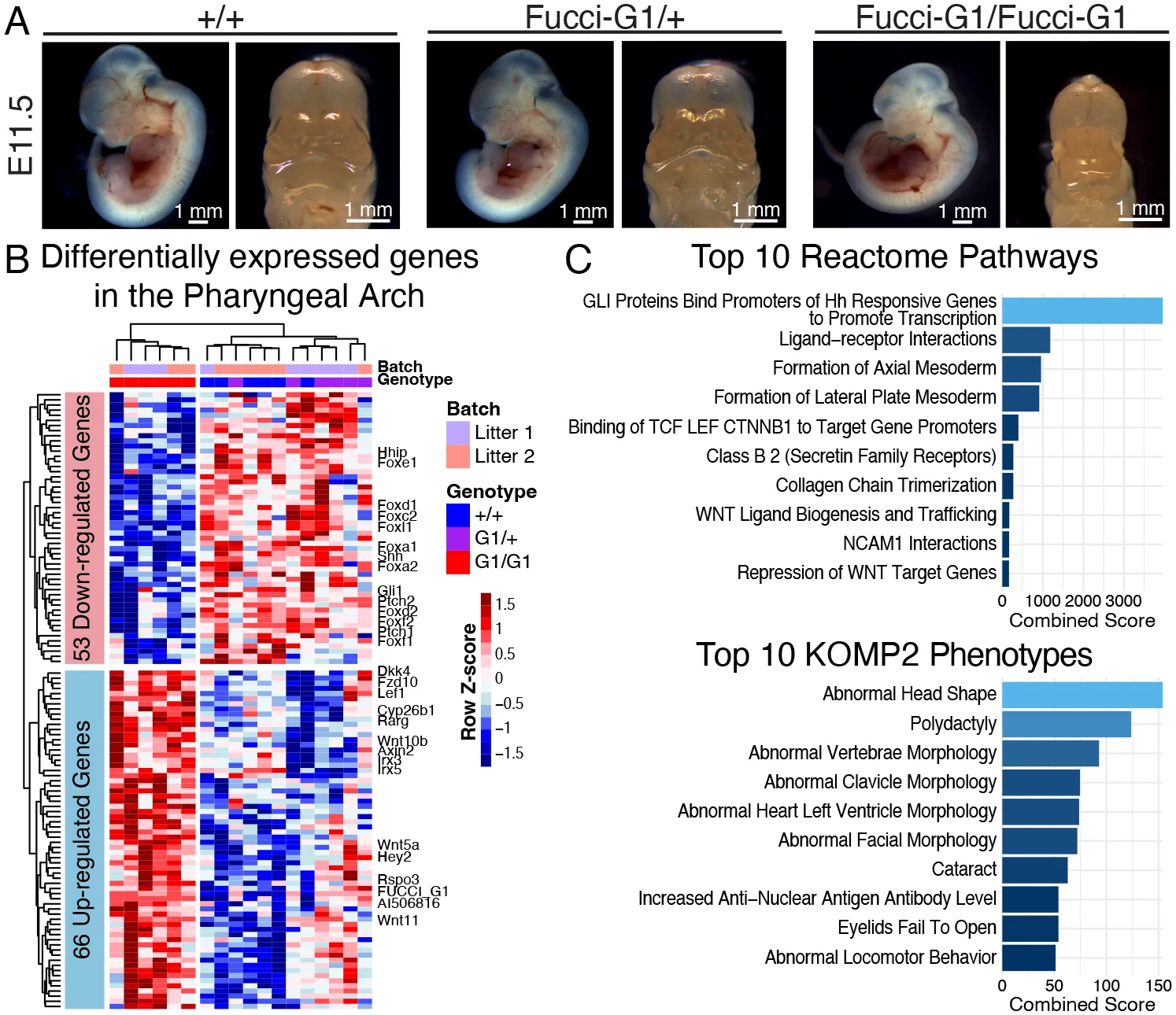
**Transcriptional profiling of E11.5 pharyngeal arches are consistent with decreased SHH signaling.** (A) Fucci-G1 homozygous embryos (Fucci-G1/Fucci-G1, right) at E11.5 display pharyngeal arch hypoplasia and abnormal craniofacial morphology compared to wild type (+/+, left) and Fucci-G1 heterozygous (Fucci-G1/+, center) littermates. (B,C) RNA sequencing of E11.5 pharyngeal arches revealed downregulation of *Shh* and SHH canonical targets (B), while we observed upregulation of the canonical and non-canonical Wnt signaling pathways, retinoic acid signaling, and Iroquois homeobox transcription factors, consistent with mis-patterning of the pharyngeal arches and craniofacial development (C). For these experiments, five homozygous, 14 heterozygous, and four wild-type embryos (A), and six homozygous, six heterozygous, and six wild-type embryos (B), were examined.

*Shh* is expressed in the pharyngeal ectoderm and foregut endoderm by E9.5 (Marti et al., 1995). Pharyngeal arch hypoplasia and abnormal craniofacial morphology is apparent at E11.5 in Fucci-G1 homozygous embryos (Fig. 3A). To analyze the role the Fucci-G1 insertion plays in arch development at the molecular level, we collected pharyngeal arches at E11.5 for tissue-level RNA sequencing (Fig. 3B). Using a multivariate approach, we identified 119 genes differentially expressed between Fucci-G1 homozygous, Fucci-G1 heterozygous, and wild type embryos [false discovery rate (FDR)<0.05; Table S1]. Strikingly, in Fucci-G1 homozygous embryos, *Shh* is downregulated 4-fold (log_2_ fold change=−1.94), while there was no difference between heterozygous Fucci-G1 embryos and controls (log_2_ fold change=−0.08). Additionally, several canonical targets of SHH were downregulated only in Fucci-G1 homozygotes (Fig. 3C), including *Ptch1* (log_2_ fold change=−0.85), *Hhip* (log_2_ fold change=−1.2), *Gli1* (log_2_ fold change=−0.55), *Foxa2* (log_2_ fold change=−1.6), and *Foxf1* (log_2_ fold change=−1.4).

Interestingly, the lincRNA, *AI506816*, located 4.7 mb downstream of *Shh*, and 4.8 mb away from the Fucci-G1 insertion site, was upregulated in the Fucci-G1 heterozygotes 6-fold (log_2_ fold change=2.5) and homozygotes 10-fold (log_2_ fold change=3.2; Fig. 3B). Although not dramatically increased or decreased, several coding genes near *AI506816* were significantly dysregulated, including *Srpk2* (log_2_ fold change=0.19 and 0.21 in Fucci-G1 heterozygous and homozygous, respectively), *Pus7* (log_2_ fold change=−0.26 and −0.39 in Fucci-G1 heterozygous and homozygous, respectively), and *Fam126a* (log_2_ fold change=−0.16 and −0.30 in Fucci-G1 heterozygous and homozygous, respectively; Table S1).

In addition to reduced *Shh* and target expression, we observed upregulation of canonical and non-canonical Wnt signaling pathways, retinoic acid signaling components, and Iroquois transcription factor members (Fig. 3B,C). Changes in these pathways are consistent with patterning defects of the pharyngeal arches and contribute to the craniofacial defects observed (Fig. 3C).

**Fig. 4.**
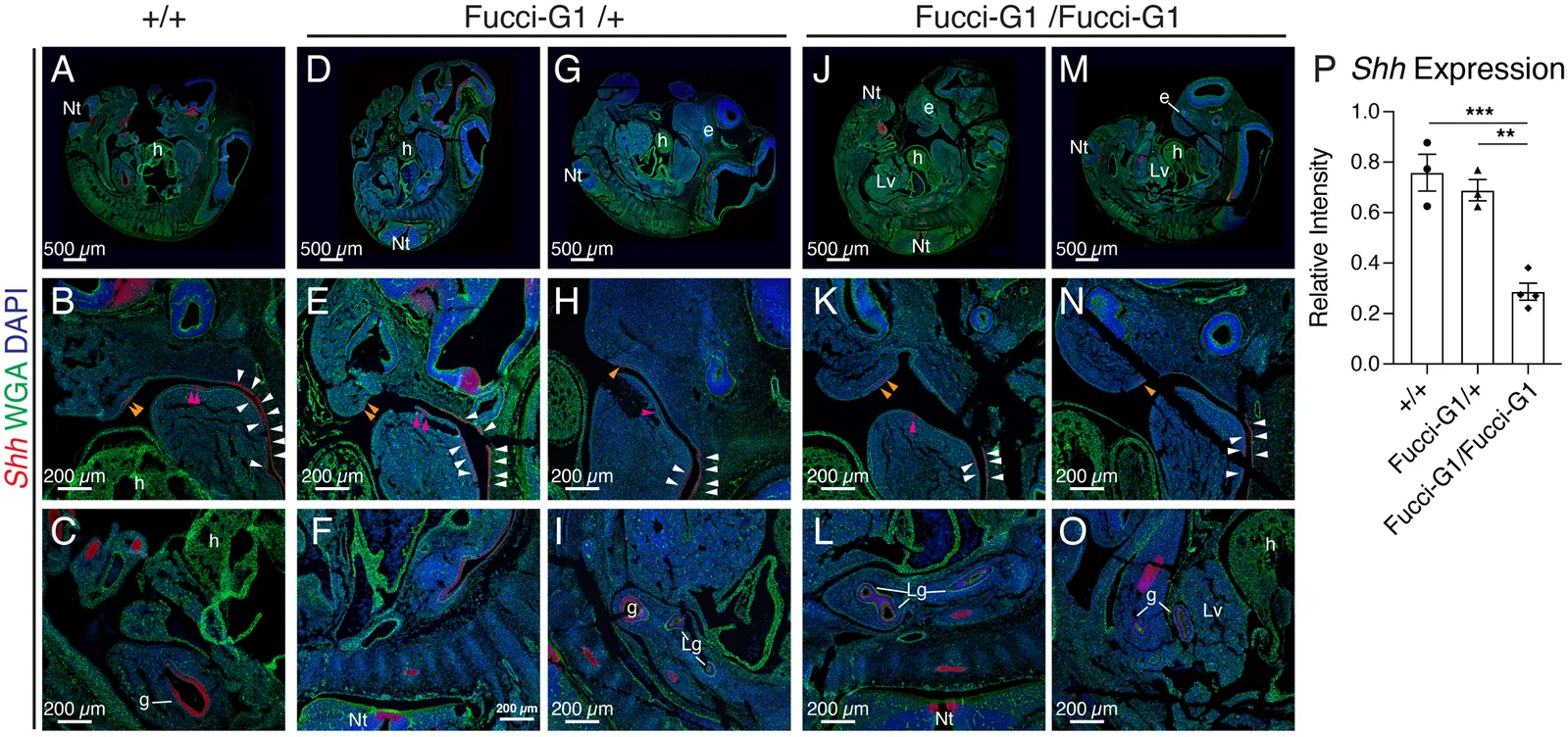
***Shh* expression is downregulated in Fucci-G1 homozygous embryos.** (A-O) RNAscope analysis of E11.5 sagittal embryo sections for wild type (+/+; A,B,C), Fucci-G1 heterozygotes (Fucci-G1/+; D-I), and Fucci-G1 homozygotes (Fucci-G1/Fucci-G1; J-O). Sections were post-stained with WGA for cell boundaries and DAPI for nuclei. (P) *Shh* levels in the foregut were compared by comparing the ratio of foregut to neural tube, and statistics were performed using a one-way ANOVA followed by a Tukey post-hoc test for multiple comparisons. For this experiment, four homozygous, three heterozygous, and three wild-type embryos were examined. e: eye, g: gut, h: heart, Lg: lung, Lv: liver, Nt: neural tube. Pink arrowheads: pharyngeal ectoderm, white arrowheads: pharyngeal endoderm, orange arrowheads: dorsal oral ectoderm. **FDR<0.01, ***FDR<0.001.

Further analysis of *Shh* expression in Fucci-G1 homozygous embryos was performed by *in situ* hybridization of E11.5 embryos and quantified (Fig. 4). Expression of *Shh* in the neural tube of Fucci-G1 heterozygous and homozygous embryos is comparable to that in wild type (Fig. 4A,D,G,J,M), working as a positive control. Expression in pharyngeal and foregut endoderm (Fig. 4B,E,H,K,N) is diminished in Fucci-G1 homozygous embryos compared to that in both wild type (FDR=5.97E-4) and Fucci-G1 heterozygous embryos (FDR=1.56E-3; Fig. 4P).

Altogether, our results show that the Fucci-G1 transgene was inserted upstream of *Shh* and disrupts tissue-specific expression of *Shh* during development, resulting in reduced jaws, lungs, and stomachs, leading to perinatal lethality in homozygous Fucci-G1 mice. The enhancer landscape of *Shh* is complex and spans a long length of DNA with several known elements regulating the various endodermal tissues (Kane et al., 2022; Anderson et al., 2014; Tsukiji et al., 2014; Sagai et al., 2009). Given the gene expression changes of *Shh* and the cluster of nearby genes, we speculate that the three-dimensional organization of this locus may be disrupted by the transgene's integration and expression, but more work needs to be done to understand this mechanism. Interestingly, *Shh* expression in heterozygous Fucci-G1 embryos was comparable to that in wild type, suggesting that dosage compensation may account for the lack of phenotype. Nevertheless, it remains possible that Fucci-G1 mice may genetically interact with SHH-related alleles in unexpected ways. We caution the use of the Fucci-G1 line in investigating SHH-related pathways.

## MATERIALS AND METHODS

### Mouse lines

Fucci-S/G2/M, B6.Cg-Tg(FucciS/G2/M)#504Bsi (Riken, RBRC02706) and Fucci-G1, B6.Cg-Tg(FucciG1)#596Bsi (Riken, RBRC02727) lines was used in this study. All mouse procedures were performed in accordance with institutional and governmental guidelines and approved by the Baylor College of Medicine Institutional Animal Care and Use Committee (Houston, TX, USA).

### Whole-genome sequencing

DNA from a compound heterozygous Fucci-G1 and Fucci-S/G2/M mouse was extracted from a tail sample using the KingFisher Flex System (Thermo Fisher Scientific) following the manufacturer's protocol. Subsequent whole-genome library preparation and sequencing was performed by Novogene Corporation, USA to a depth of approximately 30× coverage using Illumina 300 bp paired-end sequencing.

Identification of the insertion site was performed by aligning the raw sequencing reads to the mouse genome using bowtie2 (--local --very-sensitive-local --no-unal --no-mixed --no-discordant --phread33 -I 10 -X 1000 -x --un-conc-gz --al-conc; version 2.3.4.3) (Langmead and Salzberg, 2012). Unaligned and singularly aligned pairs were then ran through SPAdes (-t 16 -k 21 33 55 69 --isolate; v3.15.5) to generate *de novo* contigs (Prjibelski et al., 2020). Contigs containing each the Fucci-G1 and Fucci-S/G2/M transgenes were identified among the generated sequences. Neither contig contained sequence overlapping the mouse genome. Using the original raw FASTQ, a complete transgene was manually mapped using the contigs as a seed to extend out in both directions until reads overlapping the mouse genome was identified. In the case of Fucci-G1, this occurred at chr5:28,573,077 (terminal end) and chr5:28,593,624 (promoter end). This suggested that chr5:28,573,078-28,593,623 was lost in the insertion. In the case of Fucci-S/G2/M, we were only able to identify a single event with the sequencing performed, chr16:83,618,748 (promoter end). The generated contig contained a self-referential sequence in which the terminal end sequence of the contig started repeating the promoter end sequence before ending. One interpretation based on the limitations of the technology (Illumina 300 bp paired-end reads) and depth (∼30×) is that >1 copy of the Fucci-S/G2/M transgene may be present in tandem, but any additional copies may be incomplete resulting in a difficult to parse insertion site. The raw sequence is available at PRJNA1309140.

### Genotyping

For Fucci-G1, two separate PCR reactions are run with an annealing temperature of 60°C in both instances. The first reaction uses the following: Fucci-G1-F, 5′-GCT ACT AGA AGA TGT GCC TTT AGG AAG C-3′; and Fucci-G1-R, 5′-CTC CCA GTC ATA GCT GTC CCT CTT CTC T-3′. This produces a product of 539 bp corresponding to the Fucci-G1 transgene. The second reaction uses the following: Wild type-F, 5′-CCC TGG AAG ATA GGG AAT GA-3′; Wild type-R, 5′-AGG ACT CAC AGA GAC TAA GC-3′. This produces a product of 581 bp corresponding to the wild-type allele.

To genotype Fucci-S/G2/M, a multiplex PCR strategy was developed using the following genotyping primers: Fucci-S/G2/M, 5′-GGG CAG TTT ACC GTA AAT AGT CCA CCC A-3′; Common 5′-GCA TCC TGT GAC CCC AGT CCC TTA ATT T-3′; Wild type 5′-GTG TGA GTC TGA AGC AGT ACA-3′. For the PCR reaction, an annealing temperature of 65°C was used. This produces a wild-type allele product of 868 bp and a Fucci-S/G2/M transgene product of 433 bp.

### RNA-sequencing analysis

The pharyngeal arches of 19 E11.5 embryos were collected from two litters by microdissection and stored in TRIzol. Following genotype confirmation (six wild type, eight Fucci-G1 heterozygotes, and five Fucci-G1 homozygotes), RNA was extracted using the manufacturer's protocol. Purified total RNA was used for poly(A)-enriched, non-directional RNA-sequencing (Novogene Corporation, USA). FASTQ files were aligned to the mouse genome, mm10, using STAR (version 2.7.10a) (Dobin et al., 2013), and differential expression analysis was performed using edgeR (version 3.40.2) and limma (version 3.54.2) using genotype and litter information as covariates (Robinson et al., 2010; McCarthy et al., 2012). Significant changes were defined as an FDR less than 0.05. Pathway analysis was performed using Enrichr (Chen et al., 2013; Kuleshov et al., 2016; Xie et al., 2021). Raw and summarized sequencing data are available at Gene Expression Omnibus (GEO; GSE306148).

### *In situ* hybridization – RNAscope

E11.5 embryos were collected, fixed in 10% neutral buffered formalin, and embedded in paraffin. Samples were sectioned, and *in situ* hybridization was performed with *Shh* ACD RNAscope probes using the manufacturer’s protocol (Bio-Techne). Quantification was performed by measuring the intensity of *Shh* expression in the floor plate of the neural tube and in the pharyngeal and foregut endoderm. We then calculated the ratio to normalize pharyngeal/foregut endoderm signal and compared that using a one-way ANOVA followed by a Tukey post-hoc test for multiple comparisons.

## Supplementary Material



10.1242/biolopen.062386_sup1 Supplementary information

Table S1. Differential gene expression outputs. Columns A and B represent mouse Ensembl Gene IDs and Official Gene Symbol. Columns C, D, E, and F are the results of the differential tests with C representing log2 fold change of the Fucci G1 Heterozygote compared to WT, D representing log2 fold change of the Fucci G1 Homozygote compared to WT, and C and D are the p-value and FDR's calculated from the ANOVAlike comparison made. Columns G-X are normalized expression values for individual samples; genotype and litter number is indicated.
