# Supplementary material for: Fucci-G1 insertion partially disrupts Shh expression
Source: Biol Open. 2026 Aug 4;15(7):bio062386. doi: 10.1242/bio.062386 (PMC13488407; doi:10.1242/bio.062386)
Supplement: Supplementary file 1 [file biolopen-15-062386-s1.pdf]

Table S1. Differential gene expression outputs. Columns A and B represent mouse Ensembl Gene IDs and Official Gene Symbol. Columns C, D, E, and F are the results of the differential tests with C representing log2 fold change of the Fucci G1 Heterozygote compared to WT, D representing log2 fold change of the Fucci G1 Homozygote compared to WT, and C and D are the p-value and FDR's calculated from the ANOVA-like comparison made. Columns G-X are normalized expression values for individual samples; genotype and litter number is indicated.

Available for download at
https://journals.biologists.com/bio/article-lookup/doi/10.1242/bio.062386#supplementary-data

Biology Open • Supplementary information
