# Supplementary material for: Fucci-G1 insertion partially disrupts Shh expression
Source: Biol Open. 2026 Aug 4;15(7):bio062386. doi: 10.1242/bio.062386 (PMC13488407; doi:10.1242/bio.062386)
Supplement: Supplementary file 2 [file biolopen-15-062386_review_history.pdf]

Fucci-G1 insertion partially disrupts Shh expression

Yuka Morikawa, Ann Bromley, James F. Martin and Jeffrey D. Steimle
10.1242/bio.062386

Editor: Tristan Rodríguez

Review timeline
Original submission: 20 November 2025
Editorial decision: 27 November 2025
First revision received: 28 May 2026
Accepted: 28 May 2026

Original submission

First decision letter

MS ID#: bio.062386

MS Title: Fucci-G1 insertion partially disrupts Shh expression

Authors: Yuka Morikawa, Ann Bromley, Jeffrey D. Steimle and James F. Martin

I have now reached a decision on the above manuscript.

The reviewer reports are shown at the bottom of this email.

As you will see, the reviewers raised a number of substantial criticisms that prevent me from accepting the paper at this stage.

They suggest, however, that a revised version might prove acceptable, if you can address their concerns. If you think that you can deal satisfactorily with the criticisms on revision, I would be pleased to see a revised manuscript. We would then return it to the reviewers.

At this stage, we also ask you to ensure your manuscript complies with our formatting guidelines. Provided you are able to fully address the referees' comments, we are positive about publication of your paper (we accept over 95% of revision submissions) and therefore hope you won't mind any extra work involved in reformatting your manuscript at this point.

Please upload both a 'clean' version of your Word file, along with a highlighted version clearly showing where you have made changes in the revised manuscript. Please avoid using 'Track changes' in Word files as these are lost in PDF conversion.

I should be grateful if you would also provide a point-by-point response detailing how you have dealt with the points raised by the reviewers in the 'Response to Reviewers' box. Please attend to all of the reviewers' comments. If you do not agree with any of their criticisms or suggestions please explain clearly why this is so.

Reviewer 1

Comments for the author

© 2026. Published by The Company of Biologists under the terms of the Creative Commons Attribution License (https://creativecommons.org/licenses/by/4.0/).

This manuscript addresses the potential effects of the transgene insertion site in Fucci-G1 mice. This reporter line is widely used to monitor the cell cycle. The authors identify the insertion sites of the transgenes in both the Fucci-G1 and Fucci-S/G2/M lines. They focus on the G1 line because its insertion lies close to a Shh enhancer that regulates Shh expression in the gut and lung, leading them to hypothesise that the insertion may affect Shh transcriptional regulation during embryogenesis.

Having identified the insertion site, the authors develop a genotyping strategy and analyse morphological defects in wild-type, Fucci-G1/-, and Fucci-G1/G1 embryos. Their analysis reveals hypoplasia of the arches, lungs, stomach, and gut, which is consistent with their hypothesis. They then perform RNA-sequencing on dissected pharyngeal arches from wild-type, heterozygous, and homozygous Fucci-G1 embryos. The molecular data clearly support their model: Shh and its downstream target genes are significantly down-regulated in homozygous embryos relative to heterozygous and wild-type controls. They also report alterations in other pathways that may contribute to the observed jaw defects. Finally, they perform RNA in situ hybridisation to demonstrate reduced Shh expression in the pharyngeal endoderm and foregut of homozygous embryos, but not in heterozygous or wild-type littermates.

Overall, this is an informative manuscript that provides compelling evidence for disruption of the SHH pathway in specific endodermal tissues.

I have only a few minor comments:

Figure 3A: The frontal view is not very clear. While the lateral view nicely illustrates the hypoplasia of the branchial arches, the defects are difficult to appreciate in the frontal view. Could the authors provide alternative images that better highlight these frontal defects?

Figure 4: Additional clarification of the arrowheads would be helpful. Some appear to indicate the pharyngeal endoderm, others the pharyngeal ectoderm, and others what seems to be the dorsal oral ectoderm. These labels should be made more informative and should match the terminology used in the Results/Discussion and in the figure legend.

<u>Reviewer 2</u>

Comments for the author

In this manuscript, Morikawa et al describe the insertion sites in the genome of the transgenic constructs used to build two widely used cell cycle reporter mouse strains. The Fucci-S/G2/M strain, viable in homozygosity, has the transgene inserted at a non-distinctive location. On the other hand, the Fucci-G1 transgene is inserted nearby the well-known developmental regulatory gene Shh, and it was previously described that this strain is lethal in homozygosity. The authors show that the transgene locates only 9 kb away from a previously described cis-regulatory element that drives expression in the lung and gut (SLGE), and that its insertion caused a 20 kb deletion that includes the lincRNA Gm43161. Examination of homozygote embryos before lethality, at E18.5, showed defects in jaws, lungs, stomach and intestines. These are sites of Shh expression, and some of them show activity of the SLGE. Expression analysis at E11.5 show that in homozygous embryo there is down-regulation of Shh pathway components (by RNA-seq), and Shh expression itself is reduced in the pharyngeal ectoderm and the gut (as seen by in situ).

The data presented here, with little doubt, point to a disruption of Shh expression in the Fucci-G1 strain caused by the insertion of the transgene. Furthermore, this is possibly the cause of late embryonic lethality of homozygotes. However, this work does not address a clearly stated biological hypothesis, but just describes the observation of a transgene insertion at an interesting locus. The authors state that "The primary goal of this work is to identify the location of both transgenes to facilitate efficient breeding and genotyping of Fucci mice" (lines 45-46). However, it is not obvious nor explained how the results aid to this goal. As the line is lethal in homozygosity, but has no phenotype in heterozygosity, users will not encounter issues related to disruption of Shh when using it for cell cycle studies.

As for the description itself, some aspects could certainly be improved:

© 2026. Published by The Company of Biologists under the terms of the Creative Commons Attribution License (https://creativecommons.org/licenses/by/4.0/).

1. the images of E11.5 embryos in Fig. 3A are of very poor quality, and it is hard if not impossible to observe any clear differences in branchial arches from different genotypes.

2. including a simple table of the RNA-seq data as supplementary information would be useful.

3. RNAscope images in Fig. 4 are not detailed enough. As they show single sections, the whole pattern of expression is lost. For example, neural tube expression, that supposedly is used as a positive internal control, is limited to small patches. Expression in the lung, supposedly a site of expression of Shh affected by transgene insertion, is only shown in homozygotes, not in controls (and therefore, not mentioned in the text). Maybe other approaches, such as whole mount in situ hybridization, would show better the changes. Also, is it possible to quantify in any way these images?

4. it would be good to include heterozygous embryos in Figs 3A and 4. Also, the number of embryos analyzed in each experiment and of those showing the described phenotypes should be indicated in the figure legends.

5. The putative explanation to some of the observations as being caused by disruption to 3D chromatin structure and long-distance interactions at the locus (lines 89-91, 103-104), is purely speculative and not based on any data offered here.

Reviewer's Responses to Questions

Experimental quality
Does each figure have the proper controls?
If 'No', please indicate reasons in Comments for Author box below.
Reviewer #1:

- Yes

Reviewer #2:

- Yes

Were the data analyzed using appropriate statistical tests?
If 'No', please indicate reasons in Comments for Author box below.
Reviewer #1:

- Yes

Reviewer #2:

- Yes

Reproducibility
Were experiments performed using adequate number of biological replicates?
If 'No', please indicate reasons in Comments for Author box below.
Reviewer #1:

- Yes

Reviewer #2:

- Yes

Does the methods section provide sufficient detail to permit reproducibility?
If 'No', please indicate reasons in Comments for Author box below.
Reviewer #1:

- Yes

Reviewer #2:

© 2026. Published by The Company of Biologists under the terms of the Creative Commons Attribution License (https://creativecommons.org/licenses/by/4.0/).

- Yes

Completeness
Are the manuscript's conclusions supported by the data?
If 'No', please indicate reasons in Comments for Author box below.
Reviewer #1:
- Yes

Reviewer #2:
- No

Scholarship
Do the authors cite and discuss the merits of data that would argue for and against their conclusion?
If 'No', please indicate reasons in Comments for Author box below.
Reviewer #1:
- Yes

Reviewer #2:
- Yes

Does the manuscript title & abstract accurately reflect the contents of the manuscript, without hyperbole?
If 'No', please indicate reasons in Comments for Author box below.
Reviewer #1:
- Yes

Reviewer #2:
- Yes

First revision

Author response to reviewers' comments

Reviewer 1

1. Figure 3A: The frontal view is not very clear. While the lateral view nicely illustrates the hypoplasia of the branchial arches, the defects are difficult to appreciate in the frontal view. Could the authors provide alternative images that better highlight these frontal defects?

   We have replaced the images in Figure 3A with new higher contrast images of embryos from E11.5 that highlight the hypoplastic nature of the branchial arches among other features in the Fucci-G1 homozygous embryos.

2. Figure 4: Additional clarification of the arrowheads would be helpful. Some appear to indicate the pharyngeal endoderm, others the pharyngeal ectoderm, and others what seems to be the dorsal oral ectoderm. These labels should be made more informative and should match the terminology used in the Results/Discussion and in the figure legend.

   We have updated Figure 4 and the corresponding figure legend. We have used 3 different color arrowheads to represent the pharyngeal ectoderm (pink), pharyngeal endoderm (white), and dorsal oral ectoderm (white). We have updated the text to match to make it

© 2026. Published by The Company of Biologists under the terms of the Creative Commons Attribution License (https://creativecommons.org/licenses/by/4.0/).

clear what was quantified. We specifically quantified the pharyngeal endoderm and the rest of the foregut endoderm deeper in the embryo.

Reviewer 2

1. the images of E11.5 embryos in Fig. 3A are of very poor quality, and it is hard if not impossible to observe any clear differences in branchial arches from different genotypes

   We have replaced the images in Figure 3A with new higher contrast images that highlight the hypoplastic nature of the branchial arches of Fucci-G1 homozygous embryos.

2. including a simple table of the RNA-seq data as supplementary information would be useful

   We have now generated and added Supplemental Table 1, which includes all the outputs we generated during our RNA-seq analysis.

3. RNAscope images in Fig. 4 are not detailed enough. As they show single sections, the whole pattern of expression is lost. For example, neural tube expression, that supposedly is used as a positive internal control, is limited to small patches. Expression in the lung, supposedly a site of expression of Shh affected by transgene insertion, is only shown in homozygotes, not in controls (and therefore, not mentioned in the text). Maybe other approaches, such as whole mount in situ hybridization, would show better the changes. Also, is it possible to quantify in any way these images?

   To address the reviewer's comments, we have added more panels to Figure 4 to highlight the regions of the neural tube and lungs. We have also included Fucci-G1 heterozygous samples for comparison. Also, as the reviewer suggested, we quantified the RNAscope images. To do this, we measured the intensity of *Shh* expression in the floor plate, which appears to be robust and undisturbed by the number of Fucci-G1 transgene alleles, and compared this to the intensity of *Shh* expression in the foregut. We see both a significant difference between the normalized intensity of the wild type and Fucci-G1 homozygotes (FDR = 5.97E-4) as well as between Fucci-G1 heterozygotes and Fucci-G1 homozygotes (FDR = 1.56E-3), consistent with our RNA-seq (Figure 3).

4. it would be good to include heterozygous embryos in Figs 3A and 4. Also, the number of embryos analyzed in each experiment and of those showing the described phenotypes should be indicated in the figure legends.

   We have added matching images of littermate Fucci-G1 heterozygotes to Figures 3A and 4. To the second point, we have added the number of animals examined in each experiment to the figure legends.
   Figure 2: 3 homozygotes, 10 heterozygotes, and 6 wildtypes.
   Figure 3A: 5 homozygotes, 14 heterozygotes, and 4 wildtypes.
   Figure 3B: 6 homozygotes, 6 heterozygotes, and 6 wildtypes.
   Figure 4: 4 homozygotes, 3 heterozygotes, and 3 wildtypes.

5. The putative explanation to some of the observations as being caused by disruption to 3D chromatin structure and long-distance interactions at the locus (lines 89-91, 103-104), is purely speculative and not based on any data offered here.

   We have completely removed the original lines 89-91 and we also removed the ending of the paragraph that included lines 103-104. We have now added to the last paragraph of **Results and Discussion**: "The enhancer landscape of *Shh* is complex and spans a long length of DNA with several known elements regulating the various endodermal tissues [6-8,11]. Given the gene expression changes of *Shh* and the cluster of nearby genes, we speculate the 3-dimensional organization of this locus may be disrupted by the transgene's integration and expression, but more works needs to be done to understand this mechanism."

© 2026. Published by The Company of Biologists under the terms of the Creative Commons Attribution License (https://creativecommons.org/licenses/by/4.0/).

Second decision letter

MS ID#: bio.062386R1

MS Title: Fucci-G1 insertion partially disrupts Shh expression

Authors: Yuka Morikawa, Ann Bromley, Jeffrey D. Steimle and James F. Martin

I am happy to tell you that your manuscript has been accepted for publication in Biology Open, pending our standard publication integrity checks. It was accepted on 28th May 2026.

© 2026. Published by The Company of Biologists under the terms of the Creative Commons Attribution License (https://creativecommons.org/licenses/by/4.0/).
